# Supplementary material for: Death without Previous Hospital Readmission in Patients with Heart Failure with Reduced Ejection Fraction—A New Endpoint from Old Clinical Trials
Source: J Clin Med. 2022 Sep 21;11(19):5518. doi: 10.3390/jcm11195518 (PMC9571697; doi:10.3390/jcm11195518)
Supplement: Supplementary file 1 [file jcm-11-05518-s001.zip › Table S1.pdf]

Table S1. Supplement. Characteristics of patients group included in the heart failure trials – baseline characteristics and selected drugs

Trial	Group	Age [years]	Male sex [%]	Ischemic etiology [%]	HF duration [months]	NYHA III/IV [%]	EF [%]	HR [%]	ICD [%]	CRT-D [%]	Diuretics [%]	Beta-blocker [%]	ACE/ ARB/ ARNI [%]	MRA [%]
SOLVD-Prev	Enalapril	59.1	88.6	82.9	-	0	28	74.6	-	-	16.2	24.3	-	-
	Placebo	59.1	88.5	83.5	-	0	28	75.2	-	-	17.0	23.7	-	-
DIG	Digoxin	63.4	77.8	70.8	17	32.9	28.6	-	-	-	81.2	-	94.1	-
	Placebo	63.5	77.5	70.4	16	32.4	28.4	-	-	-	82.2	-	94.8	-
MERIT-HF	Metoprolol CR/XL	63.9	77	65	-	59.4	28	82.4	-	-	91	-	95	-
	Placebo	63.7	78	66	-	58.8	28	82.7	-	-	90	-	96	-
CHARM Alt	Candesartan	66.3	68.2	69.7	-	52.0	29.8	75.1	3.9	-	85.3	54.6	-	24.7
	Placebo	66.8	68.1	66.9	-	52.8	30.0	73.7	2.8	-	85.6	54.5	-	23.0
CHARM Added	Candesartan	64	78.8	62.2	-	75.6	28	73.4	3.7	-	90	55.0	100	17.4
	Placebo	64.1	78.6	62.6	-	76.2	28	73.7	4.2	-	90.1	55.9	99.8	16.9
CHARM	Candesartan	65.1	74.1	58.7*	-	65.1	29.0	74.1	3.8	-	87.9	54.8	55.8	20.6
	Placebo	65.3	73.9	57.8*	-	65.9	29.0	73.7	3.5	-	88.1	55.3	55.6	19.6
SENIORS	Nebivolol	76.1	61.6	68.9	-	40.5	36	79.2	-	-	85.8	-	87.9	28.8
	Placebo	76.1	64.7	67.6	-	41.0	36	78.9	-	-	85.5	-	89.7	26.4
CARE-HF	OMT + CRT	67	74	40	-	100	25	69	-	-	43	70	95	54
	OMT	66	73	36	-	100	25	70	-	-	44	74	95	59
HF-ACTION	Exercise	59.2	70.1	51.6	-	37.6	24.6	-	42.3	18.6	77.2	94.1	95.3	45.1
	OMT	59.3	73.2	51.1	-	35.7	24.9	-	38.2	17.3	78.6	94.9	93.3	45.1
HEAAL	ARB	66.0	70.0	64	-	31	33	71	-	-	77	72	77	38
	Placebo	66.0	71.0	65	-	31	33	72	-	-	76	72	76	38
MADIT-CRT	ICD + CRT	65	74.7	55.0	-	10	24	-	100	100	75.7	93.3	97.8	32.3
	ICD	64	75.6	54.9	-	10	24	-	100	0	72.9	93.2	97.2	30.9
SHIFT	Ivabradine	60.7	76	68	4.3†	52	29	79.7	3	1	84	89	93	61
	Placebo	60.1	77	67	4.2†	52	29	80.1	4	1	83	90	92	59
RAFT	ICD + CRT	66.1	84.8	68.7	-	0	22.6	-	100	100	83.6	89	97.1	41.8

	ICD	66.2	81	64.9	-	0	22.6	-	100	0	84.7	90.4	96.1	41.6
EMPHASIS	Eplerenone	68.7	77.3	69.7	4.8	0	26.2	72	13.0	5.4	84.3	86.6	94.0	100
	Placebo	68.6	78.1	68.1	4.6	0	26.1	72	13.4	7.2	85.7	86.9	92.9	0
PARADIGM-HF	Sacubitril/Valsartan	63.8	79	59.9	-	23.9	29.6	72	14.9	7	80.3	93.1	-	54.2
	Placebo	63.8	77.4	60.1	-	25.5	29.4	73	14.7	6.7	80.1	92.9	-	57.0
DAPA-HF	Dapagliflozin	66.2	76.2	55.5	-	32.3	31.2	71.5	26.2	8.0	93.4	96.9	84.5	71.5
	Placebo	66.5	77	57.3	-	32.7	30.9	71.5	26.1	6.9	93.5	96.0	82.8	70.6
VEST	Vesnarinone 130mg	62.9	75.4	56.6	52.8	99.1	20.9	-	-	-	-	-	90.4	-
	Vesnarinone 60mg	63.3	78.5	60.3	52.5	98.6	20.9	-	-	-	-	-	89.2	-
	Placebo	62.9	75.0	57.6	51.0	98.8	20.9	-	-	-	-	-	90.5	-
ELITE-2	Losartan	71.4	70	79	-	48	31	75	-	-	77	23	23	-
	Captopril	71.5	69	79	-	48	31	75	-	-	79	21	24	-
ANDROMEDA	Dronedarone	71	74.2	66.5	20	57.8	-	78	1.3	-	92.9	61.9	88.4	42.3
	Placebo	72	76.3	63.4	23	61.8	-	81	1.9	-	95.3	60.6	84.2	39.1
GISSI-HF P	PUFA	67	77.8	49.1	-	36.3	33.0	72	7.1	-	89.5	65.1	93.5	38.6
	Placebo	67	78.8	50.3	-	36.8	33.2	73	7.2	-	90.0	64.6	93.4	40.0
GISSI-HF S	Rosuvastatin	68	76.2	39.8	-	38.8	33.4	73	6.4	-	90.0	62.7	94.1	39.0
	Placebo	68	78.6	40.2	-	36.1	33.1	73	6.8	-	90.0	62.0	92.9	41.3
ECHOS	Nolomirole	70	73	-	-	55	-	81	-	-	-	49	89	55
	Placebo	70	74	-	-	55	-	79	-	-	-	49	87	50
ACCLAIM	Immunomodulation therapy	64.6	80	68	-	70	22.7	72	26	10	94	87	94	48
	Placebo	64.0	80	69	-	73	22.6	72	26	11	94	87	94	51
ASCEND-HF	Nesiritide	67	66.6	59.5	-	-	-	82	-	-	94.9	57.4	59.7	27.5
	Placebo	67	65.1	60.8	-	-	-	82	-	-	95.3	58.9	61.8	28.2
ECHO-CRT	CRT	57.6	72.8	54	-	96.3	27.0	-	-	100	85.6	95.8	94.8	61.1
	Control	58.3	71.9	53	-	97.8	27.0	-	-	-	86.9	97.5	94.8	58.8
RED-HF	Darbepoetin Alfa	72	59.7	73.2	3.8†	67.4	31.0	72	-	-	90.5	85.4	87.9	43.6
	Placebo	71	57.4	72.6	3.5†	63.0	30.0	71	-	-	92.5	84.7	89.9	45.8
ASTRONAUT	Aliskiren	64.7	78.8	64.4	-	63	27.9	77.9	15.6	6.8	95.9	81.7	84.9	55.4

	Placebo	64.5	75.6	62.8	-	66	27.8	77.9	15.7	6.7	95.8	83.4	83.6	58.6
ATMOSPHERE	Combination therapy	63.2	78.9	57.1	-	36.0	28.5	72	-	-	79.9	92.0	-	36.6
	Aliskiren	63.3	77.3	55.3	-	35.9	28.4	72	-	-	79.1	91.2	-	36.9
	Enalapril	63.3	78.6	55.7		38.3	28.3	72	-	-	80.4	91.9	-	37.8
COMMANDER HF	Rivaroxaban	66.5	78.0	-	-	52.0	35	-	-	-	-	-	-	-
	Placebo	66.3	76.2	-	-	53.7	34	-	-	-	-	-	-	-

* - history of MI;

† – data available in years

HF - heart failure, EF – ejection fraction; HR – heart rhythm, ICD - implantable cardioverter-defibrillator; CRT - cardiac resynchronization therapy; ACEI – angiotensin-converting enzyme inhibitor; ARB - angiotensin receptor blockers; ARNI - angiotensin receptor-neprilysin inhibitor; MRA – mineralocorticoid receptor antagonist;
