# Supplementary material for: Death without Previous Hospital Readmission in Patients with Heart Failure with Reduced Ejection Fraction—A New Endpoint from Old Clinical Trials
Source: J Clin Med. 2022 Sep 21;11(19):5518. doi: 10.3390/jcm11195518 (PMC9571697; doi:10.3390/jcm11195518)
Supplement: Supplementary file 1 [file jcm-11-05518-s001.zip › Tables S2-S8.pdf]

Table S2. The **death from CV causes** or HF hospitalization / HF worsening endpoint – death from CV causes without hospital admission for HF / worsening HF

Trial	Group	n	Follow-up	Composite endpoint		Hospital admission for HF		CV death		CV death without hospital admission for HF		
			Months	N	%	n	%	n	%	n	%	p
FOLLOW-UP 12-24 MONTH												
DAPA-HF	Dapagliflozin	2373	18.2	382	16.1%	231	9.7%	227	9.6%	151	6.4%	0.14
	Placebo	2371		495	20.9%	318	13.4%	273	11.5%	177	7.5%	
EMPHASIS	Eplerenone	1364	21	249	18.3%	164	12.0%	147	10.8%	85	6.2%	0.19
	Placebo	1373		356	25.9%	253	18.4%	185	13.5%	103	7.5%	
COMMANDER	Rivaroxaban	2507	21.1	932	37.2%	689	27.5%	453	18.1%	243	9.7%	0.78
	Placebo	2515		929	36.9%	691	27.5%	476	18.9%	238	9.5%	
SHIFT	Ivabradine	3241	22.9	793	24.5%	514	15.9%	449	13.9%	279	8.6%	0.45
	Placebo	3264		937	28.7%	673	20.6%	491	15.0%	264	8.1%	
EMPEROR	Epaglizflozin	1863	16	361	19,4%	246	13,2%	187	10,0%	115	6,2%	0.28
	Placebo	1867		462	24,7%	362	19,4%	202	10,8%	100	5,4%	
FOLLOW-UP 24-36 MONTH												
PARADIGM-HF	Sacubitril/Valsartan	4187	27	914	21.8%	537	12.8%	558	13.3%	377	9.0%	0.004
	Placebo	4212		1117	26.5%	658	15.6%	693	16.5%	459	10.9%	
CHARM Altern.	Candesartan	1013	33.7	334	33.0%	207	20.4%	219	21.6%	127	12.5%	0.62
	Placebo	1015		406	40.0%	286	28.2%	252	24.8%	120	11.8%	
ATMOSPHERE	Combination	2340	36.6	770	32.9%	430	18.4%	512	21.9%	340	14.5%	
	Aliskiren	2340		791	33.8%	442	18.9%	562	24.0%	349	14.9%	0.71<br>0.51
	Enalapril	2340		808	34.5%	452	19.3%	457	19.5%	356	15.2%	
FOLLOW-UP > 36 MONTHS												
CHARM	Candesartan	2289	40	817	35.7%	516	22.5%	521	22.8%	301	13.1%	0.96
	Placebo	2287		944	41.3%	642	28.1%	599	26.2%	302	13.2%	
CHARM Added	Candesartan	1276	41	483	37.9%	309	24.2%	302	37.0%	174	13.6%	0.63
	Placebo	1272		538	42.3%	356	28.0%	347	41.2%	182	14.3%	

Table S3. The **all-cause death** or HF hospitalization / HF worsening endpoint – death for any reason without hospital admission

Trial	Group	n	Follow-up	Composite endpoint		Hospital admission for HF		All-cause death		All-cause death without hospital admission for HF		
			Months	n	%	n	%	n	%	n	%	p
FOLLOW-UP < 6 MONTHS												
ASCEND-HF *	Placebo	3413		345	10.1%	208	6.1%	141	6.9%	137	4.0%	0.19
	Nesiritide	3423	1.0	321	9.4%	204	6.0%	126	5.8%	117	3.4%	
ANDROMEDA	Placebo	317		40	12.6%	30	9.5%	12	3.8%	10	3.2%	0.11
	Dronedarone	310	2.0	53	17.1%	35	11.3%	25	8.1%	18	5.8%	
FOLLOW UP 6-12 MONTHS												
VEST	Placebo	1283		382	29.8%	237	18.5%	242	18.9%	145	11.3%	
	Vesnarinone 30mg	1275	9.4	395	31.0%	232	18.2%	268	21.0%	163	12.8%	0.25
	Vesnarinone 60mg	1275	9.5[†]	410	32.2%	217	17.0%	292	22.9%	193	15.1%	0.04
ACCLAIM	Placebo	1204		265	22.0%	210	17.4%	128	10.6%	55	4.6%	0.27
	IMT	1204	10.2	292	24.3%	221	18.4%	117	9.7%	71	5.9%	
MERIT-HF	Placebo	2001		439	21.9%	294	14.7%	217	10.8%	145	7.2%	0.03
	Metoprolol CR/XL	1990	12	311	15.6%	200	10.1%	145	7.3%	111	5.6%	
FOLLOW-UP 12-24 MONTHS												
ECHO CRT	CRT	404	19.4	116	28.7%	99	24.5%	45	11.1%	17	4.2%	0.34
	Control	405		102	25.2%	90	22.2%	26	6.4%	12	3.0%	
EMPHASIS	Eplerenone	1364	21	270	19.8%	164	12.0%	171	12.5%	106	7.8%	0.26
	Placebo	1373		376	27.4%	253	18.4%	213	15.5%	123	9.0%	
COMMANDER	Rivaroxaban	2507	21.1	993	39.6%	689	27.5%	546	21.8%	304	12.1%	0.31
	Placebo	2515		973	38.7%	691	27.5%	556	22.1%	282	11.2%	
EMPEROR	Epaglizflozin	1863	16	407	21,8%	246	13,2%	249	13,4%	161	8,6%	0.5
	Placebo	1867		512	27,4%	362	19,4%	266	14,2%	150	8,0%	

FOLLOW-UP 24-36 MONTHS												
MADIT-CRT	CRT + ICD	1089	28.8	187	17.2%	151	13.9%	74	6.8%	36	3.3%	0.3
	ICD	731	28.8†	185	25.3%	167	22.8%	53	7.3%	18	2.5%	
CARE-HF	OMT + CRT	409	29.4	118	28.9%	72	17.6%	82	20.0%	46	11.2%	0.43
	OMT	404		191	47.3%	133	32.9%	120	29.7%	58	14.4%	
FOLLOW-UP >36 MONTHS												
SOLVD Pre	Enalapril	2111	37.4	434	20.6%	184	8.7%	313	14.8%	250	11.8%	0.79
	Placebo	2117		518	24.5%	273	12.9%	334	15.8%	245	11.6%	
CHARM	Candesartan	2289	40	910	39.8%	516	22.5%	642	28.0%	394	17.2%	0.54
	Placebo	2287		1020	44.6%	642	28.1%	708	31.0%	378	16.5%	
RAFT	CRT + ICD	894	40	297	33.2%	174	19.5%	186	20.8%	123	13.8%	0.81
	ICD	904		364	40.3%	236	26.1%	236	26.1%	128	14.2%	
SOLVD	Enalapril	1285	41.1	613	47.7%	332	25.8%	452	35.2%	281	21.9%	0.48
	Placebo	1284		736	57.3%	470	36.6%	510	39.7%	266	20.7%	
HEAAL	Losartan 150	1921	56.4	828	43.1%	450	23.4%	635	33.1%	378	19.7%	0.7
	Losartan 50	1913	56.4†	889	46.5%	503	26.3%	665	34.8%	386	20.2%	

* – different numbers of patients reached the endpoint; † – follow-up was provided in other units than months

Table S4. The **all-cause death** or CV hospitalization – death for any reason without CV hospital admission

Trial	Group	n	Follow-up	Composite endpoint		Hospital admission for CV		All-cause death		All-cause death without hospital admission for CV		
			Months	n	%	n	%	n	%	n	%	p
ACCLAIM	IMT	1204	10.2	399	33.1%	375	31.1%	117	9.7%	24	2.0%	<0.0001
	Placebo	1204		429	35.6%	356	29.6%	128	10.6%	73	6.1%	
SENIORS	Nebivolol	1067	21	332	31.1%	256	24.0%	169	15.8%	76	7.1%	0.064
	Placebo	1061		375	35.3%	276	26.0%	192	18.1%	99	9.3%	
CARE-HF	OMT + CRT	409	29.4	159	38.9%	125	30.6%	82	20.0%	34	8.3%	0.43
	OMT	404		224	55.4%	184	45.5%	120	29.7%	40	9.9%	
HEAAL	Losartan 150	1921	56.4*	1037	54.0%	762	39.7%	635	33.1%	275	14.3%	0.49
	Losartan 50	1913		1085	56.7%	826	43.2%	665	34.8%	259	13.5%	

* – follow-up was provided in other units than months

Table S5. The **CV death** or CV hospitalization – death for any reason without CV hospital admission

Trial	Group	n	Follow-up	Composite endpoint		Hospital admission for CV		CV death		CV death without hospital admission for CV		
			Months	n	%	n	%	n	%	n	%	
SENIORS	Nebivolol	1067	21	305	28.6%	256	24.0%	123	11.5%	49	4.6%	0.019
	Placebo	1061		350	33.0%	276	26.0%	145	13.7%	74	7.0%	
EPHESUS	Eplerenone	3319	16	885	26.7%	606	18.3%	407	12.3%	279	8.4%	0.0006
	Placebo	3313		993	30.0%	649	19.6%	483	14.6%	344	10.4%	

Table S6. The **all-cause death** or all-cause hospitalization – death for any reason without any hospital admission

Trial	Group	n	Follow-up	Composite endpoint		Hospital admission for any reason		All-cause death		All-cause death without any hospital admission		
			Months	n	%	n	%	n	%	n	%	p
ACCLAIM	IMT	1204	10.2	557	46.3%	519	43.1%	117	9.7%	38	3.2%	0.27
	Placebo	1204		534	44.4%	505	41.9%	128	10.6%	29	2.4%	
MERIT HF	Metoprolol CR/XL	1990	12	641	32.2%	581	29.2%	128	6.4%	60	3.0%	0.002
	Placebo	2001		767	38.3%	668	33.4%	203	10.1%	99	4.9%	
EPHESUS	Eplerenone	3319	16	1730	52.1%	1493	45.0%	478	14.4%	237	7.1%	0.003
	Placebo	3313		1829	55.2%	1526	46.1%	554	16.7%	303	9.1%	
HF-ACTION	Exercise	1159	30	759	65.5%	729	62.9%	189	16.3%	30	2.6%	0.48
	Usual Care	1172		796	67.9%	760	64.8%	198	16.9%	36	3.1%	

Table S7. The **CV death** or all-cause hospitalization – death for CV reason without any hospital admission

Trial	Group	n	Follow-up	Composite endpoint		Hospital admission for any reason		CV-cause death		CV death without any hospital admission		
			Months	n	%	n	%	n	%	n	%	p
GISSI-HF PUFA	n-3 PUFA	3494	46.8*	2157	61.7%	1986	56.8%	712	20.4%	171	4.9%	0.84
	Placebo	3481		2202	63.3%	2028	58.3%	765	22.0%	174	5.0%	
GISSI-HF STATIN	Rosuvastatin	2285	46.8*	1417	62.0%	1278	55.9%	478	20.9%	139	6.1%	0.007
	Placebo	2289		1385	60.5%	1286	56.2%	488	21.3%	99	4.3%	

* – follow-up was provided in other units than months

Table S8. The **death** related to HF / HF worsening or hospitalization due to HF / HF worsening – death for HF worsening without any hospital admission for HF worsening

Trial	Group	n	Follow-up	Composite endpoint		Hospital admission for HF worsening		Death due to HF worsening		Death due to HF worsening without hospital admission for HF worsening		
			Months	n	%	n	%	n	%	n	%	p
EMPHASIS	Eplerenone	1364	21	170	12.5%	164	12.0%	45	3.3%	6	0.4%	0.45
	Placebo	1373		262	19.1%	253	18.4%	61	4.4%	9	0.7%	
DIG	Digoxin	3397	37	1041	30.6%	910	26.8%	394	11.6%	131	3.9%	0.19
	Placebo	3403		1291	37.9%	1180	34.7%	449	13.2%	111	3.3%	
